# An Optimal Transport Based Transferable System for Detection of Erroneous Somato-Sensory Feedback from Neural Signals

**DOI:** 10.3390/brainsci11111393

**Published:** 2021-10-23

**Authors:** Saugat Bhattacharyya, Mitsuhiro Hayashibe

**Affiliations:** 1 School of Computing, Engineering and Intelligent Systems, Ulster University, Magee Campus, Londonderry BT48 7JL, UK; 2 Department of Robotics, Tohoku University, Sendai 980-8579, Japan; mitsuhiro.hayashibe@inria.fr; 3 Department of Biomedical Engineering, Tohoku University, Sendai 980-8579, Japan

**Keywords:** brain–computer interfacing, error related potential, functional electrical stimulation, somato-sensory feedback, optimal transport, transfer learning

## Abstract

This study is aimed at the detection of single-trial feedback, perceived as erroneous by the user, using a transferable classification system while conducting a motor imagery brain–computer interfacing (BCI) task. The feedback received by the users are relayed from a functional electrical stimulation (FES) device and hence are somato-sensory in nature. The BCI system designed for this study activates an electrical stimulator placed on the left hand, right hand, left foot, and right foot of the user. Trials containing erroneous feedback can be detected from the neural signals in form of the error related potential (ErrP). The inclusion of neuro-feedback during the experiments indicated the possibility that ErrP signals can be evoked when the participant perceives an error from the feedback. Hence, to detect such feedback using ErrP, a transferable (offline) decoder based on optimal transport theory is introduced herein. The offline system detects single-trial erroneous trials from the feedback period of an online neuro-feedback BCI system. The results of the FES-based feedback BCI system were compared to a similar visual-based (VIS) feedback system. Using our framework, the error detector systems for both the FES and VIS feedback paradigms achieved an F1-score of 92.66% and 83.10%, respectively, and are significantly superior to a comparative system where an optimal transport was not used. It is expected that this form of transferable and automated error detection system compounded with a motor imagery system will augment the performance of a BCI and provide a better BCI-based neuro-rehabilitation protocol that has an error control mechanism embedded into it.

## 1. Introduction

Brain–computer interfaces (BCIs) have led to numerous advances in neuro-rehabilitation by providing a communication and control channel that bypasses the muscular activation of the limbs and relies more on the intention of the users as decoded from their neural activities. This technology was initially conceived for the benefit of patients with neural disorders, such as post-stroke effects, amyotrophic lateral sclerosis, spinal injuries, and physical disabilities [1,2], but as research has progressed in this area so has the potential applications of this technology in communications [3], automation [4], the military [5], and gaming [6]. Electroencephalography (EEG) is the most commonly used modality for the recording of neural signals [7,8]. Although the potential for applying BCI systems in clinical and general applications is high, such an approach remains extremely demanding in terms of the cognitive attention and effort required [9]. Even after numerous years of research, BCI systems are still prone to error in the detection of mental intentions, making them unreliable for real-world applications.

The reliability of a BCI can be improved by detecting errors made by the system and by including an error-correction process to rectify the previous erroneous decision. One such way is the detection of error-related potentials (ErrPs) directly from the neural signals of the users when controlling the system [10,11,12]. Earlier studies on EEG signals related to erroneous actions during a speed response task have been reported [13,14,15]. ErrP signals are usually generated when a subject commits an error or observes an error being committed either by another user or an agent [16]. Hence, an ErrP signal is usually generated while the user performs another task, such as when using a P300 speller or a motor/mental imagery system, and the system provides erroneous feedback (in visual, auditory, or sensory form, among others) to the user. The choice of feedback depends on the intended application of the BCI [17].

Numerous researchers have aided in the design of automatic ErrP detectors, which have been successfully implemented in BCIs involving sensori-motor rhythms and in event-related potentials (ERPs) such as the P300. Early research on ErrPs was reported by Ferrez et al. [10], in which an ErrP was used as a corrective measure to cancel the movement of the cursor for an incorrectly classified motor intention. In addition, one of the first studies on integrating ErrP-based correction on an online P300 speller system was reported in [18], in which the authors detected a single trial ErrP with an accuracy of approximately 60%. In addition, Schmidt et al. [19] used an online ErrP recognition system in their Center Speller [20]. A similar system was used by Spüler et al. [21] on young, elderly, and motor-impaired individuals with ALS and Duchenne muscular dystrophy. Bevilacqua et al. [22] explored the possibility of detecting ErrPs during a pseudo-online motor imagery BCI speller task. Studies also exist that showed no or little improvement of BCI performance after using ErrP for automatic error correction [18,23], or the participants were more confused and preferred not to use BCI with the error correction system [23]. When implementing an automatic error detector using ErrP, it is expected that the classification performance of ErrP will be higher to that of the companion BCI signal (such as, P300, motor imagery, etc); otherwise, the overall performance will suffer. It can be attributed to small datasets with even less occurrence of ErrP. Recent studies have aimed to improve the detection of ErrP by using double detection of single-trial responses [24,25], or by implementing more robust classification techniques [26]. An extensive review of the applications of ErrPs for motor-impaired individuals along with briefs on existing challenges and future direction can be found in [27,28].

An open area of research prevalent to a BCI is the need for the design of a zero-training or minimal-training system that can completely remove or reduce the need for constantly training users before each new session. This requirement arises from the non-stationarity found in an EEG owing to changes in the electrode location and impedances [29], as well as changes in the cognitive state of the user [30]. Transfer learning approaches are being extensively investigated for this purpose. Current approaches applied for cross-subject transfer learning include a least squares transformation of the source EEG [31], k-nearest neighbor [32], and multi-subject common spatial patterns [33,34]. Some deep-learning approaches have also been proposed using adversarial networks and manifold constraints for cross-subject classification [35,36,37]. Extensive details on transfer learning approaches applied to a BCI are discussed in [38,39].

In our previous study [40], we used functional electrical stimulation (FES) [41,42] as a form of neuro-feedback to motor-imagery BCI tasks. FES is traditionally used for stroke rehabilitation, and operates by directing electrical stimulation to the muscles located in the impaired section of the body, and aims at eliciting a recovery of daily life skills, such as standing, grasping, cycling, and walking, by re-training the users regarding these tasks [43,44]. In [40], we demonstrated that FES-based feedback augments the motor-learning skills of the participants. In this study, we aim to detect a response evoked in the brain signals of the participants in the form of ErrP when they observe (in the case of visual feedback) or sense (in case of FES as feedback) an erroneous trial. Such feedback could be due to either the participant or the online classifier making an error. Erroneous perception is a frequently occurring cognitive process in our daily life. The motor imagery paradigm is popularly employed in BCI for neuro-rehabilitation. Thus, verifying erroneous perception from neural signals while the user is performing a primary task (for example, motor learning of upper limbs) is an important issue. In a previous study [45], a reinforcement learning based BCI was developed that uses the ErrP signals to control the activation of an FES device. In the present study, we first detect whether individuals trained only on motor imagery tasks can identify incorrect feedback by eliciting ErrPs. If detected, we aim to study the effects of FES on such elicitation and compare the results with standard visual feedback. If successful, this detection of incorrect feedback will allow patients to directly intervene in their motor recovery process and will make the neuro-rehabilitation paradigm more interactive and reliable to them. This study marks the first time such an approach has been undertaken.

In this experiment, the participants underwent training not to evoke an ErrP and for only motor imagery tasks. Hence, no preliminary data on the participants were available that could be used to train the error detector. Thus, as a work-around, we have developed a cross-subject transferable, automated ErrP detection system for individual participants by considering the knowledge representation of other participants carrying out the same tasks. In this study, we apply optimal transport theory [46] as a transfer learning technique to train a classifier on erroneous and correct trials for a known group of users and to test it on an unknown user (cross-subject). An optimal transport was previously used in source localization using an EEG/MEG [47], P300 [48], and sleep stage detection [49], although this is the first time it is being used for error detection.

The rest of this paper is as follows: Section 2 briefly describes the experiment protocol adopted for the study of FES as a type of neuro-feedback during a motor-imagery BCI task. This section also provides insight into the proposed method adopted for an automated ErrP detection system based on transfer learning. Section 3 provides a detailed discussion on the results and their significance. A summary of this study and future approaches including potential areas of application are discussed in Section 4, followed by some concluding remarks in Section 5.

## 2. Materials and Methods

**Figure 1:**
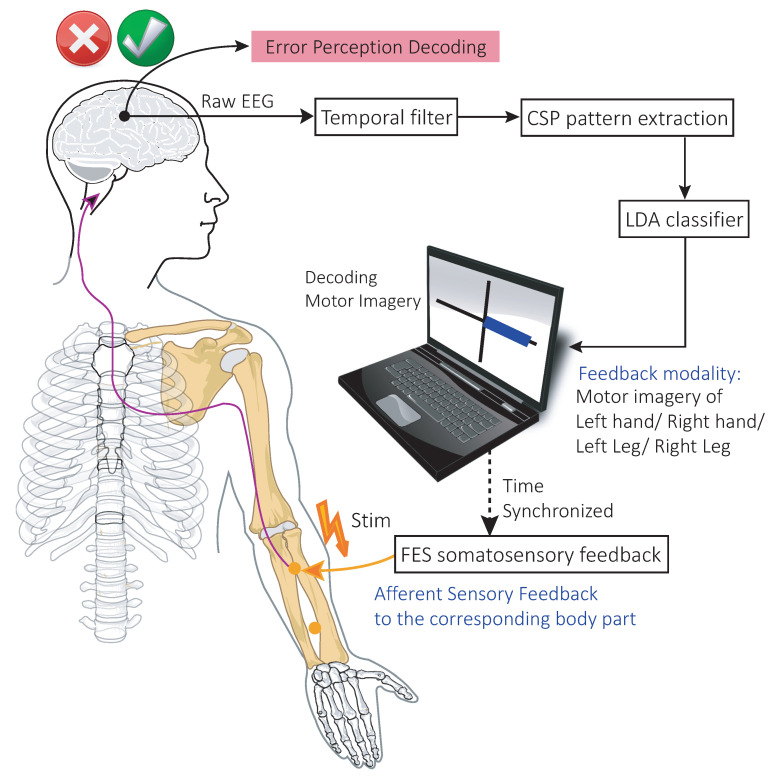
Representation of the online BCI system for the VIS and FES groups, as described in Section 2. Based on the LDA classifier output, the occurrence of ErrP signals is expected to be detected from incorrectly classified trials (as mentioned above).

As FES feedback is tactile in nature, it is quite possible that the participants will elicit ErrP when the wrong limb receives the feedback. ErrP signals are generally identified by a negative deflection occurring at 50–100 ms after the feedback response, which is immediately followed by a positive peak at approximately 200–500 ms after such a response [50]. The positive peak is due to the conscious post-error adjustment made by the participant [9]. The complete flow of the online experiment conducted by the FES and VIS groups and a conceptualised diagram of detecting erroneous feedback during the online experiment is illustrated in Figure 1. In this study, we designed an automatic, transferable error detector tuned to detect ErrP signals. In its current iteration, the system is applied offline, but, in future experiments, an online version will be implemented to assist the participants with an error correction. Section 2.1, Section 2.2, Section 2.3 and Section 2.4 briefly describe the online neuro-feedback BCI experiment using functional electrical stimulation as a feedback modality that we conducted for a four-class motor imagery class. Extensive details of the experiment are provided in [40]. Then, from Section 2.5 onward, we describe our methodology in extracting characteristic features from erroneous and correct feedback and the implementation of a transferable decoder to correctly predict those feedback across participants.

### 2.1. Data Acquisition

The neural signals were recorded using a TMSI Refa8 EEG amplifier at a sampling rate of 256 Hz from 17 electrode locations in the fronto-central, central, centro-parietal, and parietal regions (arranged in a standard 10–20 configuration), namely, Fz, FC3, FCz, FC4, C5, C3, C1, Cz, C2, C4, C6, CP3, CPz, CP4, P3, Pz, and P4. The left ear mastoid was used as a reference electrode while the right ear electrode was used as the ground electrode.

The surface electrical stimulation was delivered using a computer-controlled stimulator (ProStim, MXM, France) with a maximum pulse width (PW) modulation [51] of 400 μs at a constant amplitude and frequency (20 Hz). Each stimulation sequence consisted of a trapezoidal envelope train PW (0.4 s ramp-up, 1.2 s plateau, and 0.4 s ramp-down). Rectangular electrodes of 5 × 9 cm in size were placed in the anterior compartment of the upper-limbs and the triceps surae muscle group for the lower-limbs to induce a wrist flexion and foot plantar flexion, corresponding to the mental task performed. The electrical stimulation was set by the participants before the start of the experiment to a comfortable level. Nevertheless, it was ensured that the electrical stimulation did not exceed 25 mA.

The EEG recording, display of visual cues, feedback, and online classification of the motor imagery tasks were conducted using OpenVIBE software [52]. The output of the classifier was sent as a command to the stimulator through MATLAB. An offline analysis for automated ErrP detection was conducted in a Python 3.5 environment, the results of which are discussed in Section 3.

### 2.2. Participants

Sixteen naive participants (13 male and 3 female, with a mean age = 28 ± 9 years) volunteered for this study. Among the 16 participants, 8 received feedback regarding the electrical stimulation (henceforth, called the FES group), whereas the other 8 were provided visual feedback (henceforth, called the VIS group). The selection of participants in the FES and VIS groups was conducted at random. The participants undertook the experiment in isolation while sitting in a comfortable chair placed in front of a display monitor placed at eye level. The subjects were informed about the purpose of the experiment and the tasks they would be required to perform prior to the start of the experiment. After this debriefing, if they agreed to continue with the experiment, the participants signed an informed consent form that was previously approved by the institutional (Inria) ethical committee. Following the completion of the experiment, the participants were asked to provide a subjective assessment of how focused they were while performing the tasks on scale of 1–−10.

### 2.3. Task Description and Visual Cue

The participants performed the cued motor imagery tasks using their left hand, right hand, left foot, and right foot. Each participant from the FES and VIS groups underwent a single training session followed by three feedback sessions. Each session consisted of 24 trials for each motor imagery task.

**Figure 2:**
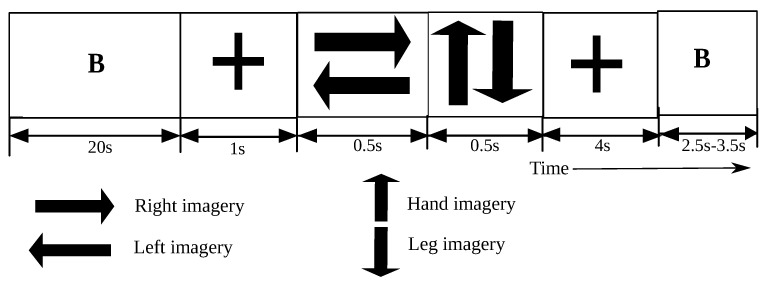
Sequence of visual cues displayed to the participants during the online neuro-feedback experiment. Each trial began with the display of a fixation cross, denoted by ‘+’ in the figure, followed by a left or right arrow indicating the left or right side of the body, and finally an up or down arrow to indicate whether to move the hand or foot. The second ‘+’ is the feedback period, which is the time interval of interest in this study.

The visual cues (Figure 2) were displayed in the following sequence. At the beginning of each trial, a fixation cross was displayed on the screen for 1 s. Motor imagery instructions were then provided for 1 s in the form of arrows. The sequence of arrows was as follows: a left/right arrow to indicate a motor imagery on the left/right side, followed by an up/down arrow to indicate whether to move the hand or foot. For example, if a right arrow was displayed followed by a down arrow, then the participant must imagine moving the right foot. The instruction cues were followed by a feedback period of 4 s, where the participant conducted the task at hand and had the chance to visualize (VIS group)/sense (FES group) their own performance. There was delay of 500 ms in the projection of the feedback (that is, the display of a feedback bar or the relay of electrical stimulation) owing to the computations involved by the online decoder. Finally, after the feedback period, a blank screen was displayed for 2.5–3.5 s, allowing the participants to relax.

### 2.4. Generation of Online Motor Imagery Commands for Feedback

While the participants were conducting the experiment, continuous streams of EEG signals were recorded using Openvibe software. The continuous EEG signals were segmented into lengths of 5 s (*epochs*), starting from 1 s before the onset of the feedback period of each motor imagery (MI) task. Each epoch was then filtered using a fourth-order Butterworth band-pass filter at [.5,40] Hz, notch filtered at 50 Hz and re-referenced at Fz. Hence, the influence of muscle artefact, electrical stimulator noise, and line noise were removed at this stage of the processing. Finally, log-transformed variances of the first and last spatial filters were computed using common spatial patterns (CSPs) [53] to extract the feature vectors. These feature vectors were used as inputs to a multi-class linear discriminant analysis (LDA) classifier [54].

The classifier (in Openvibe) predicted the outcome of the motor imagery task conducted by the participant during each trial in the form of output labels and the hyperplane distance. The feedback begins to be elicited 500 ms after the start of the feedback period and is constant for the whole duration of the feedback period. The FES group receives feedback to the limb (right/left hand or right/left foot) corresponding to the output label at different intensities of the stimulation pulse widths, depending on the hyperplane distance. The intensity of the electrical stimulation does not go beyond that selected by the participant at the beginning of the experiment. The feedback for the VIS group was provided to the subject in the form of a blue bar. If the bar appeared to the right of the fixation cross, the participant was informed that the classifier had achieved a correct prediction. Otherwise, for an incorrect classification, the bar appears to the left of the fixation cross. It must be noted that the FES group received no form of visual feedback. We had observed that there were no discriminable features from the EEG signals of the left and right foot movement which was further reflected in the classification accuracy. Hence, for our offline studies on this dataset, we had combined both the right and left foot imagery as one that is foot imagery.

### 2.5. Filtering and Processing of EEG Signals

First, the recorded EEG signals were segmented into epochs within the range of [0, 1.5] s, where 0 indicates the onset of the feedback period. As mentioned earlier, the feedback would be sensed (for the FES group) or visualized (for the VIS group) 500 ms after the onset of the feedback. Thus, our signal of interest, that is, the ErrP, should be present after 500 ms. The epochs were then low-pass filtered at a pass band of 0-6 Hz and a stop band of 8–128 Hz with an optimal finite impulse response (FIR) filter designed using a Remez exchange algorithm [55]. We kept the higher cut-off at 6 Hz to remove any overlapping of the motor imagery signals with our signal of interest. ErrP signals are dominant in the range of [0.1, 10] Hz [10] while motor imagery signals are dominant in the range of [8, 12] Hz [40]. As the online experiment is based on motor imagery control, some overlap between motor imagery and ErrP waveforms were found in electrode Cz during preliminary analysis. As the focus of analysis in this study is to detect whether ErrP were being generated within the participants during the online experiment, a passband of [0–6] Hz was selected in this study to limit the influence of motor imagery signals in the classification process (while taking into consideration an attenuation of the signal amplitude).

Based on the literature [27], ErrP signals are dominant in the anterior cingulate cortex of the human brain. Thus, we selected FCz, Cz, and CPz electrodes for analysis because they are the closest to this region. A surface Laplacian filter [56] was applied to the EEG signals of these electrodes to spatially filter the signal (for example, to spatially filter Cz, the mean of the adjoining electrodes C1, FCz, C2, and CPz were subtracted from the original signal at Cz). The spatially filtered signals were then baseline corrected using signal segments retrieved from 300 ms before the onset of the motor imagery stimulus. We chose this earlier segment of the signal for a baseline correction to avoid contamination from any signals occurring from the motor imagery EEG signals. Finally, the processed signal was down-sampled by a factor of 16. The resulting down-sampled signal of length 24 was then used as the features for each channel. The feature vector prepared has dimensions of Number_of_Trials×(features×channels)=96×(24×3)=96×72.

### 2.6. Relabeling of Epochs to Correctness

To detect erroneous trials in the form of ErrP signals, we re-labeled the trials with correct feedback (produced by the online BCI) as ‘*correct*’, whereas the trials with incorrect feedback were labeled as ‘*incorrect*’. The percentage of correct and incorrect feedback for each participant is shown in Figure 4a,b (see Section 3.1). Henceforth, all analyses described in this paper use ‘correct’ and ‘incorrect’ trials as the ground truth.

### 2.7. Transfer Learning Using Regularized Discrete Optimal Theory

Intuitively speaking, transport theory is the study of optimal solutions to transport mass between two probability distributions by minimizing the transportation cost. Optimal transport was first formulated by Gaspard Monge in 1781 as a resource allocation problem that searches for a transport map to minimize a certain cost. Kantorovic [57] proposed an adaptation of the optimal transport problem that looks for a probabilistic coupling to minimize the cost function. Recent studies have begun modifying and implementing an optimal transport to solve covariate shifts [46] in domain adaptation problems. Extensive details on optimal transport for domain adaptation can be found in [46,58]. In this study, we briefly describe this method for ErrP detection across different participants. The methodology applied here was adopted from an earlier study on a P300 Speller classification problem [48].

#### 2.7.1. Background

Let us consider $V={\left\{\left(f_{i},c_{i}\right)\right\}}_{i=1}^{N}$ to be the data obtained from a participant or volunteer. Each participant has undergone *N* trials, and the feature vectors $F={f_{i}}_{i=1}^{N}\subset R^{d}$ of dimension *d* have corresponding classes $C={c_{i}}_{i=1}^{N}$. Assume that we have a group of participants as *sources* and another group as *targets*. Their corresponding neural data will then be denoted as $V^{s}$ and $V^{t}$. Let us assume that the classes for the sources are known and the targets are to be estimated. In addition, being a transfer learning problem, it is assumed that the source and target domains have been subject to a covariate shift. Here, we aim to recover a transport plan between the probability distribution of the source domain $P(F_{s})$ and the target domain $P(F_{t})$. This plan will allow us to map the *source* domain onto the *target* domain, and a classifier trained on the *transported source* data can finally predict the classes of the *target* data.

The discrete adaptation of our problem is limited to the matching of empirical measures $\mu_{s}$ of $P(F_{s})$ and $\mu_{t}$ of $P(F_{t})$ owing to a fixed number of samples (trials). The empirical distribution μ for both the source and target domain are given by the following:

(1) $$\mu =\sum_{i=1}^{N}p_{i}\delta_{f_{i}}$$

where $p_{i}$ is the probability mass (associated with either the *source* or *target*), and $\delta_{f}$ is the Dirac distribution for feature *f*. Considering $p^{s}$ and $p^{t}$ as the probability mass of the *source* and *target* data and $1_{N}$ as a unit vector of the I-dimension, we can then compute the transport plan $\tau_{0}$ in such a manner that, when probabilistic couplings occur between $\mu_{s}$ and $\mu_{t}$, $X=\{\tau \in {\left(R^{+}\right)}^{N_{s}\times N_{t}}|\tau^{s}1_{N^{t}}=p^{s},\tau^{t}N_{I^{s}}=p^{t}\}$, and thus $\tau_{0}\in X$ can be derived from the following minimization problem:

(2) $$\begin{array}{cc} \tau_{0}= & {\mathrm{argmin}}_{\tau \in X}<\tau ,J{>}_{F}+\\ \; & \lambda \sum_{i,j}\tau \left(i,j\right)\log\tau \left(i,j\right)+\\ \; & \eta \sum_{j}\sum_{c}l||\tau \left(I_{c},j\right){\left|\right|}_{2} \end{array}$$

where <.> is the Frobenius dot product, and $I_{\rfloor }$ represents a set of indices corresponding to class c∈{correct,incorrect}.

The first term of Equation (2) represents the discrete adaptation of the Kantorovic formulation [57], and *J* denotes the cost function matrix, which is actually the cost required to move the probability mass from $f_{i}^{s}$ to $f_{j}^{t}$. The squared Euclidean distance given by $d\left(f_{i}^{s},f_{j}^{t}\right)=\left|\right|f_{i}^{s}-f_{j}^{t}{\left|\right|}^{2}$ is the preferred metric for a unique coupling, and we therefore use it in the present study.

The second term of Equation (2) is the first regularization term that solves the optimization problem using the Sinkhorn–Knopp algorithm [59]. The third term of the equation, proposed by Courty et al. [46], is a regularizer that ensures that new samples will give mass only to existing samples of the same class by inducing a group-sparse penalty on the columns of $\tau_{0}$. In this study, we set the regularization value to 10.

Finally, the new location of the *target* data is computed using barycentric mapping $\cap F^{s}=\mathrm{diag}{\left(\tau_{0}^{T}1_{N_{s}}\right)}^{-1}\tau_{0}^{T}F^{t}$, where $\cap F^{s}$ and $F^{t}$ are the feature vectors of the transported *source* and *target* data, respectively.

#### 2.7.2. Classification between Correct and Incorrect Trials

**Figure 3:**
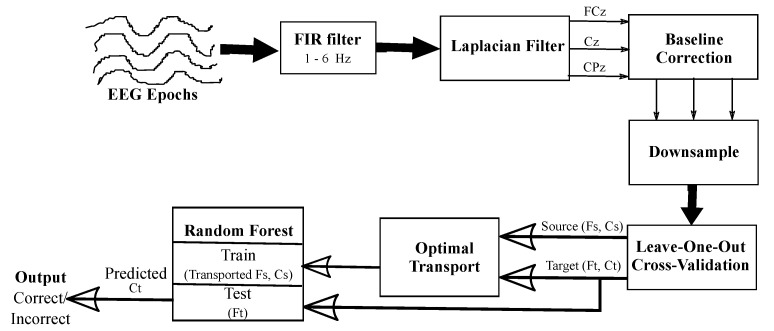
Block diagram representing the error detection pipeline, beginning from the processing of neural signals to the generation of the feature vectors, the transport of the source data to the target domain, and, finally, classification using random forest.

The formulation of the optimal transport problem allows the source features to transport to the target domain whose labels are unknown to the classifier. The next step is to train a classifier on the *transported source* features to predict the unknown *target* features. In our study, we used the *leave-one-out* cross validation method to split the feature vectors of individual participants into training and test sets. In each fold of this method, the optimal transport and classification processes will be tested on one participant, while being trained on the remaining participants. Each training set is used to train the optimal transport and classification algorithm on the correct and incorrect trials, which is then used to predict the trials for the corresponding test set. An illustration of the transport of features and class prediction during training and testing is provided in Figure 3.

Herein, we trained a random forest model [60] to achieve our goal of ErrP detection. We had employed grid-search to find the optimal parameters for our study. We found that the model which had 100 decision trees applied bootstrapping (samples are drawn with a replacement during training) and employed the *Gini* criterion, yielding the best result. The random forest approach fits sub-samples (with a replacement) of the dataset on various individual decision trees and the final output is an average of the individual results obtained from each decision tree. This form of estimation improves the prediction accuracy and controls an over-fitting. With the use of stratified cross-validation, we further ensured that our results did not benefit from an over-fitting. The metrics used to evaluate our proposed methodology are discussed in the next section.

### 2.8. Classification Metrics

In this study, the performance of the automatic ErrP detector has been evaluated based on the precision, recall, and F1-score [61]. *Precision* represents the ratio of correctly classified positive predictions (in our case, ‘incorrect’ classes) over all positive predictions. *Recall* highlights the ratio of correctly classified positive classes that were predicted correctly to the actual number of positive classes. *F1-score*, given as 2×(Recall×Precision)/(Recall+Precision), seeks to balance the precision (ratio of correctly predicted positive observations to the total number of positive observations predicted) and recall (ratio of correctly prediction positive observations to all observations in the actual class) by reducing the numbers of false positives and false negatives. Hence, it is an effective performance measure for a dataset with an uneven class distribution and has a distinct advantage over the accuracy metric.

## 3. Results

### 3.1. Online Feedback Accuracy for FES and VIS Groups

**Figure 4:**
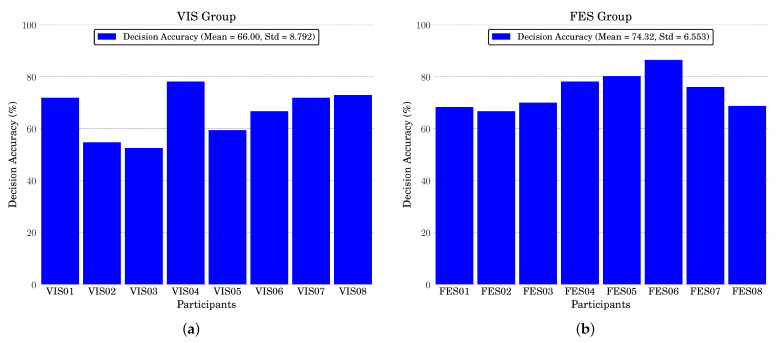
Accuracies of the online feedback of eight participants from the (**a**) VIS and (**b**) FES groups while conducting an online motor imagery feedback task.

Figure 4a,b show the accuracy of the online feedback of the individual participants during the motor imagery tasks. It was observed that the eight participants in the VIS group were correct for 66% (Standard deviation = 8.792) of all trials, with participant VIS04 achieving an accuracy of 78.125%, whereas the eight participants in the FES group were correct for 74.32% of all tasks (Standard deviation = 6.553) with participant FES06 achieving the highest accuracy of 86.458%.

### 3.2. Analysis of Event-Related Potentials for Correct and Incorrect Trials

**Figure 5:**
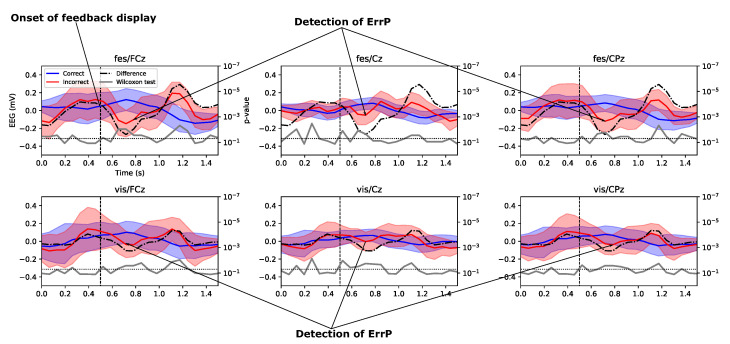
Grand averages of the event-related potentials at electrodes FCz, Cz, and CPz during correct (in blue) and incorrect (in red) feedback received while the participants in the FES (**top panel**) and VIS (**bottom panel**) groups were conducting the motor imagery tasks. The differences between the correct and incorrect ERP signals are plotted in black, and the corresponding *p*-values of the Kruskal–Wallis test [62] at each time instance are compared between the correct and incorrect feedback trials. The black-dotted horizontal line represents the 5% significance level, and the dotted vertical line represents the onset of a feedback display (both visual and somato-sensory) received by the participants.

Figure 5 shows the grand averages of the ERPs with a 1.5 s length from the onset of the feedback period at electrodes FCz, Cz, and CPz. The grand averages were computed using all participants in the FES (top panel) and VIS (bottom). One of the aims of this study was to detect whether feedback from erroneous trials elicits ErrP signals among the participants. As mentioned earlier, the participants received feedback 500 ms after the onset of the feedback period (marked by the dotted line vertical in the figure). Thus, for an ERP analysis, we focused on the period 500 ms after the onset of the feedback period.

The ERPs show consistent negative peaks (albeit with differing magnitudes) in all electrodes at approximately 200–300 ms after the onset of the feedback display (as shown in the figure, 700–800 ms after the beginning of the feedback period) followed by a positive peak. The difference in signal between the correct and incorrect feedback (in black) further magnifies the presence of positive and negative peaks. Furthermore, the profile of the *p*-values of the Kruskal–Wallis significance tests (in grey, twin-*y*-axis) shows a statistically significant difference between the correct and incorrect feedback at the negative and positive peaks which validates the presence of ErrPs for incorrect trials. The ERPs also show a statistically significant difference before the onset of the feedback display, particularly for the Cz electrode. This may be interpreted as the presence of some lingering effects related to the motor imagery tasks, and a further investigation will be required in this regard.

It must also be noted that the differences in amplitude of the ERPs for the FES group are higher than those for the VIS groups. It is expected that this phenomenon will be highlighted in the classifier performance, and it can be considered that FES feedback also evokes a greater response in detecting error than visual feedback.

### 3.3. Classification Results from Optimal Theory Based Transfer Learning

In this section, we present the classification performance in the form of the precision, recall, and F1-score for both FES and VIS groups. First, we compare the results obtained with and without the use of an optimal transport in our classification pipeline. Next, we describe the results for the FES and VIS groups using the optimal transport and random forest (as a classifier). Finally, we compare the performance of our random forest (RF) classifier with other commonly used classification algorithms. A two-tailed Wilcoxon signed-rank test [63] was employed for all comparisons.

#### 3.3.1. Comparison of Decoder Performance with and without Optimal Transport

Table 1 and Table 2 show the performances of the individual participants in the FES and VIS groups, respectively, for our proposed pipeline using an optimal transport and random forest classifier. For comparison, we designed another classification pipeline that does not employ an optimal transport process. Here, we used a *leave-one-out* cross validation method (similar to the one mentioned in Section 2.7.2) to split the feature vectors of the individual participants into training and test sets. In each fold of the cross-validation, the classifier (without the optimal transport process) was tested on one of the participants while being trained on the remaining participants. The results of this pipeline are shown in Table 3 and Table 4.

The results in the tables indicate a significant improvement in the performance when the optimal transport is employed. The average precision, recall, and F1-score in the FES group (Table 1 and Table 3) significantly improve by 29.92%, 19.43%, and 28.5%, respectively (p<0.0118 for all metrics). A similar improvement of 26.12% (p<0.0118 for all metrics) is also noted for the average precision, recall, and F1-score in the VIS group (Table 2 and Table 4).

#### 3.3.2. Comparison of Decoder Performance between FES and VIS Feedback

The previous section clearly shows that optimal transport enhances the performance of the error decoder. Furthermore, in Table 1 and Table 2, we can see that the average performance of the FES group is significantly superior to that of the VIS groups, i.e., 9.12% in terms of precision, 9.17% in terms of recall, and 9.56% for the F1-score (p<0.05 for all metrics). The results validate our claim made in Section 3.2, and it can be concluded that the higher amplitude of N1 and P1 observed in the FES groups are reflected in the higher classification results.

#### 3.3.3. Comparing Decoder Performance with Other Machine Learning Algorithms

Table 5 compares the average F1-score of our random forest classifier with other commonly used machine learning algorithms, including a linear discriminant analysis (LDA), logistic regression, linear support vector machines (SVMs), bagging ensemble classifier with LDA as a weak learner, and an Adaboost classifier. The Python package *scikit-learn* was used for classification.

The following parameters (after grid search) were employed for each of the comparative algorithms:

- **LDA:** Solver=least square (‘lsqr’), shrinkage=automatic based on Ledoit-Wolf lemma.
- **Logistic Regression:** L2 regularization = $10^{-3}$ (C = 1000), tolerance = $10^{-4}$, solver = ‘lbfgs’ (limited-memory Broyden-Fletcher-Goldfarb-Shannon algorithm), and maximum iteration for convergence = 100.
- **SVM:** Penalization norm = ‘l2’, loss function = ’hinge’, tolerance = $10^{-4}$, regularization parameter C = 1.0, and maximum iteration for convergence = 1000.
- **Bagging:** Base estimator = same as the LDA mentioned earlier, number of estimators = 100, and bootstrap = True.
- **Adaboost:** Base estimator = a decision tree classifier with a maximum depth of 1, number of estimators = 100, and learning rate = 1.0.

The classification pipeline for all methods is preceded by the optimal transport process of transporting the source samples to the target domain. It can be seen from the table that the RF classifier is superior to the rest of the classifiers but significantly better than only the FES group against the LDA, Bagging, and Adaboost classifiers (p<0.0175). The results indicate that it is possible to combine an optimal transport with other machine algorithms with a further optimization of the parameters. The reason for selecting random forest in our study is not limited to its better performance but also includes its ability to avoid an over-fitting, robustness to small changes in the dataset, encouragement of diversity, and interpretability of the results.

## 4. Discussion

The online experiment described in this paper was originally designed to study the effects of functional electrical stimulation on the motor learning of the participants. A few questions arose from this experiment and some of these questions were answered in this study.

From our online experiment, we had observed that more than 25% of the trials displayed incorrect feedback (see Figure 4a,b). Thus, when the participants observed or sensed an incorrect feedback, did they evoke some form of ErrP during the feedback period, and if so, did the FES feedback have an influence in a manner similar to the motor learning? In addition, the participants underwent training sessions to perform the motor learning neuro-feedback tasks. No such training was given to the participants for error detection, and hence the classifier will not have training samples for a given participant. Can we therefore design a transferable, automatic classification approach to detect error feedback from neural signals?

The presence of characteristic ErrP peaks in the grand-averages of the incorrect feedback trials (see Figure 5) answered our first question. The larger amplitudes of the negative and positive ErrPs in the FES feedback groups suggest that ErrPs generated in the FES group were more prevalent than those in the VIS group. This assumption is further vindicated by the performance of our offline transferable ErrP decoder approach. The classification results (Table 1 and Table 2) of the FES group were significantly better than those of the VIS group. The superior classification results compounded with the larger ErrP peaks suggest that sensory feedback through FES is more effective in eliciting an ErrP than the standard visual feedback, which answers our second question. The subjective assessment (see Section 2.2) of the participants also indicated that they took a more focused approach toward the task when they were provided with FES feedback rather than VIS feedback, which is a probable reason for the superior performance of the FES group.

**Figure 6:**
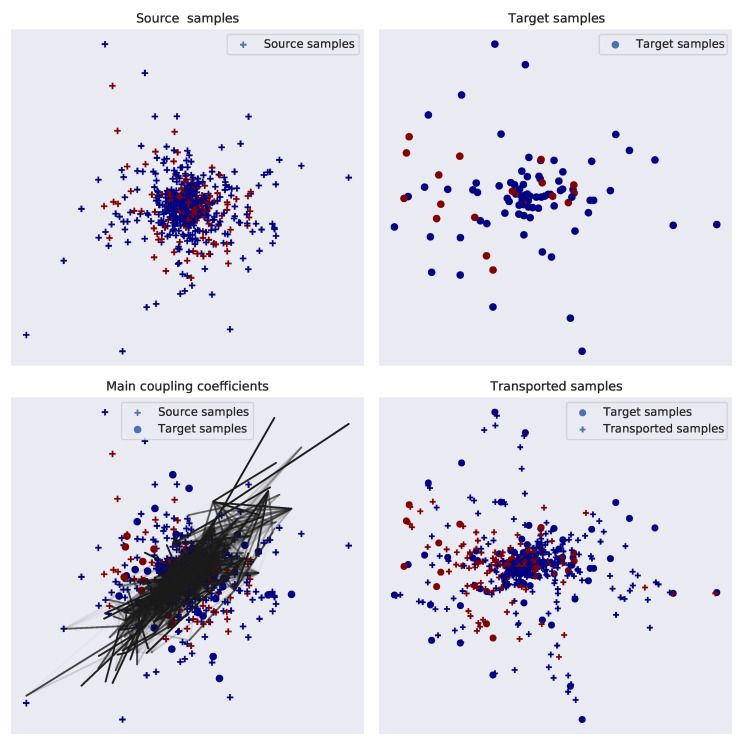
An illustration describing the transport of the probability distribution from the source domain to the target domain. The transport mapping in this example is obtained from Subject 5 of the FES group for the first and last features of the feature vector. The top two panels show the distribution of the source and target domains, followed by a probabilistic coupling between the two domains (bottom-left panel), and finally in the bottom-right panel, the distribution of transported sources is mapped together with the targets. The ‘+’ and ‘o’ mark the source (original and transported) and target samples, and the red and blue markers represent features associated with incorrect and correct trials, respectively.

We also found that our ErrP decoder performed significantly better than a similar decoder but without using optimal transport theory for the transfer learning (see Table 2 and Table 3). To understand why this is so, Figure 6 provides an example of the optimal mapping. First, as the figure indicates, the distribution of the source (here, training dataset) differs from the target (test dataset) (the top panel in the figure). Upon incorporating the optimal learning, the source samples are coupled with the target samples (see the bottom-right panel of the figure), and the transported source samples are shown (in the bottom-right panel) to adopt the distribution pattern of the target samples for both the *correct* and *incorrect* classes. This migration of the source samples to a new feature space led to a significant improvement of the classifier performance. This method can also be adopted by other classification algorithms, as shown in Table 5. Our proposed methodology has the ability to adapt to the changing dynamics of the neural signals across sessions and participants and can automatically detect ErrPs without any prior training (of a user), hence meeting the requirements of our third research question.

A previous study [45] had successfully controlled an FES system using BCI while employing an ErrP for taking corrective measures. The study compared the performance of the ErrP decoder between a control (healthy) subject and a SCI patient. The performance of our ErrP decoder is better than the performance reported in the study. We also designed our error detection methodology to be transferable to other users with no prior training sessions which was not the case of the earlier work. Moreover, we went a step further in our study and showed the positive effects of FES feedback on detecting errors that in turn helped augment the classifier performance. Our future study on BCI based-FES rehabilitation will incorporate such an automatic error detectors to help augment the motor learning experience of patients by taking necessary corrective measures as quickly as possible. Without such error-correction mechanism, it is possible for patients to get demoralized when they get incorrect feedback which might lead them to abandon this rehabilitation technology. Thus, the addition of an automatic and transferable error detection system may improve the confidence of the patient while improving the retention of the rehabilitative technology.

Even though our transferable ErrP detector showed significant improvement in performance, the study was still implemented in an offline setting. To implement this approach in an online setting, we will design an asynchronous form of BCI-based error monitoring system that will be added along with the motor imagery BCI system. The error monitoring system will begin monitoring the EEG signals from the anterior cingulate cortex at an interval of 150–300 ms (the final interval will be determined after more research) from the onset of the neuro-feedback period. On detection of error, the error monitoring system will automatically shut the neuro-feedback and prompt the participant to re-do the trial one more time.

Furthermore, the optimal transport algorithm employed in this study was semi-supervised in nature because labels of the training and test datasets were used for optimal mass movement (but not during the classification stage). In our online setting, we will employ an unsupervised form of the optimal transport algorithm. In our future studies, we will first aim to improve the existing transferable framework including unsupervised training to design a more robust and adaptable ErrP decoder. This framework has been tested for only this problem, but results from our present study and previous studies [47,48,49] shows the efficiency of implementing optimal transport for transferable EEG decoding. Nevertheless, we will continue testing our transferable error detection approach in more motor-related, cognitive and behavioural experiments so that we can develop a more generalised error detection framework.

Lastly, the experiment only considered FES and VIS feedback and did not account for a control group of participants who were given no feedback. Moreover, we will make changes to our stimuli paradigm and incorporate foot motor imagery as an overall individual class rather than using right and left foot motor imagery separately. We will also design experiments more realistic in nature and with better control conditions to improve the practicality of the current BCI. If experiments on healthy participants are successful, then we will aim to validate the effectiveness of combining BCI and FES along with our error monitoring system on patients undergoing physical rehabilitation and compare its efficacy with the current state-of-the-art.

## 5. Conclusions

This study provided conclusive evidence regarding the presence of ErrP signals on the EEG of participants conducting motor imagery tasks while receiving feedback in the form of electrical stimulation. The detection of ErrP allows the participants to correct their movements while taking necessary action to continue activating the wrong limb (i.e., the limb that is not of interest). Our transferable, offline error decoder can successfully identify at least 85% of incorrect trials. The superior error detection achieved when using FES feedback suggests that it will be a suitable alternative to visual feedback in rehabilitative applications. The incorporation of corrective measures in the cortical learning process of BCI combined with the peripheral learning process of FES would further augment the motor recovery process of patients undergoing neuro-rehabilitation by providing an opportunity to patients to directly intervene and correct their actions which will in turn increase the confidence of patients towards this technology for frequent and longer use.

## Figures and Tables

**Table 1:** Classifier performance (in %) of FES group with optimal transport.

	Precision	Recall	F1-Score
**FES01**	96.98	96.67	96.71
**FES02**	93.33	93.33	93.33
**FES03**	94.98	95.00	94.96
**FES04**	84.98	85.42	85.15
**FES05**	88.72	87.50	87.93
**FES06**	100.00	100.00	100.00
**FES07**	93.08	91.67	91.97
**FES08**	92.57	91.67	91.26
**Mean**	93.08	92.66	92.66
**SD**	4.35	4.43	4.43

**Table 2:** Classifier performance (in %) of VIS group with optimal transport.

	Precision	Recall	F1-Score
**VIS01**	87.36	87.50	86.98
**VIS02**	86.65	85.94	85.74
**VIS03**	77.40	76.25	75.80
**VIS04**	95.25	95.31	95.25
**VIS05**	87.63	84.37	83.42
**VIS06**	76.24	76.04	76.13
**VIS07**	80.61	81.25	80.76
**VIS08**	80.54	81.25	80.73
**Mean**	83.96	83.49	83.10
**SD**	5.97	5.92	5.94

**Table 3:** Classifier performance (in %) of FES group without optimal transport.

	Precision	Recall	F1-Score
**FES01**	46.69	68.33	55.48
**FES02**	78.53	68.33	57.05
**FES03**	49.00	70.00	57.64
**FES04**	65.33	75.00	68.51
**FES05**	73.14	79.17	73.96
**FES06**	89.08	87.50	82.56
**FES07**	57.63	75.00	65.18
**FES08**	45.83	62.50	52.88
**Mean**	63.16	73.23	64.16
**SD**	15.06	7.23	9.68

**Table 4:** Classifier performance (in %) of VIS group without optimal transport.

	Precision	Recall	F1-Score
**VIS01**	49.18	60.94	54.43
**VIS02**	48.38	51.56	46.25
**VIS03**	56.50	53.75	42.52
**VIS04**	77.49	79.69	77.96
**VIS05**	56.36	59.38	52.51
**VIS06**	51.08	61.46	53.58
**VIS07**	61.28	69.79	62.17
**VIS08**	62.50	70.83	63.54
**Mean**	57.84	63.42	56.62
**SD**	8.91	8.81	10.44

**Table 5:** Combining optimal transport with other existing classifiers.

	Average F1-Score	Average F1-Score
	FES	VIS
**LDA**	85.66	79.43
**Logistic Regression**	90.57	81.34
**SVM**	90.60	81.22
**Bagging**	85.91	80.15
**Adaboost**	85.61	77.81
**RF**	92.66	83.10

## Data Availability

The data are available on request to the corresponding author.
